# A review of strategies and levels of community engagement in strengths-based and needs-based health communication interventions

**DOI:** 10.3389/fpubh.2024.1231827

**Published:** 2024-04-09

**Authors:** Jesse Stover, Laxmisupriya Avadhanula, Suruchi Sood

**Affiliations:** ^1^Department of Community Health and Prevention, Drexel University Dornsife School of Public Health, Philadelphia, PA, United States; ^2^Johns Hopkins Center for Communication Programs, Department of Health Behavior and Society, Bloomberg School of Public Health, Baltimore, MD, United States

**Keywords:** health communication, community engagement, strengths-based approaches, needs-based approaches, consensus strategies, conflict strategies, global health

## Abstract

**Background:**

Community engagement is key in health communication interventions that seek to incorporate community voices in their planning and implementation. Understanding what approaches and strategies are currently being used can help tailor programs in different social and cultural contexts. This review explores needs-based and strengths-based approaches and consensus and conflict strategies in community-based global health communications programs. Our objective is to examine the current state of the field, outline lessons learned, and identify gaps in existing programming to help guide future interventions.

**Methods:**

PubMed and Web of Science were searched for articles published between 2010 and 2023. Studies were included if they described a community-based health communication intervention and an ongoing or completed implementation. Interventions were coded then categorized according to their level of community engagement and as single, hybrid, or complex, depending upon the number of approaches and strategies used.

**Results:**

The search yielded 678 results and 42 were included in the final review and analysis. A vast majority 34 (81.0%) interventions utilized a needs-based approach and 24 (57.1%) utilized a strengths-based approach. Consensus as a strategy was utilized in 38 (90.5%) of the manuscripts and 9 (21.4%) implemented a conflict strategy. Interventions that combined approaches and strategies were more likely to leverage a higher level of community engagement.

**Conclusion:**

These results showcase the complicated nature of global health communication program planning and implementation. There is a lack of interventions that use conflict as a strategy to empower communities to act on their own behalf, even when at odds with existing power structures. Complex interventions that include all approaches and strategies demonstrate the potential for global health communication interventions to be at the cutting edge of public health practice.

## 1 Introduction

### 1.1 Defining a community

Public health communication interventions are often designed with the objective of promoting social and/or individual change. However, reaching and changing behavior one person at a time is often impractical, resulting in the question: “Where is the public in public health?” Community level interventions are intended to reach groups of people at the same time. It is important to remember that such interventions may target changes at the individual level (e.g., increasing awareness or changing behavior), or changes at other levels of the social ecological model, but use a community as their base.

The term community can mean different things. It can be defined by a geographical location, such as a block, neighborhood, or city. A community can also consist of a group of people with a shared identity (such as the LBGT community), a shared interest (such as the vegan community), or shared beliefs (like a religious community). A community can also be a group of people that are linked by communication media. This is important when considering digital communities where people do not live in the same geographic location but are linked via websites and the Internet. Community health is a specific area of public health focused on the health of the people who are part of a community (1).

### 1.2 Community participation and engagement

Community participation and engagement has long been a cornerstone for public health communication interventions. An example of an early, large-scale, community-based intervention in the U.S. was the Stanford Three Community Study in 1972, later scaled up as the Stanford Five-City Project in 1980. This community-based intervention was designed to test if a comprehensive program would produce significant changes in cardiovascular disease risk factors. Directed at all residents of several cities, the intervention used mass media and interpersonal communication for the public, as well as health professionals, to build institutional and societal support for change (2). Research results showed significantly greater improvements in knowledge of cardiovascular disease, blood pressure, and smoking in the cities that received the community-based intervention, compared to a control city. Research conducted years later found that the public health benefits of the Stanford study were laid in accelerating positive risk factor change by including effective, context-specific models and strategies at the community level. This provided the groundwork for future collaboration through individual, community, and policy efforts (3).

The North Karelia Project is another intervention comparable to the Stanford Three Community Study that demonstrated the importance of context-specific community participation (4). This far-reaching project sought to decrease the excessively high cardiovascular mortality rate among men in Finland’s easternmost province, Karelia. The intervention included large-scale community organizing, like discussing diet at community events and holding training seminars with local health care workers, community members, and caterers. Originally meant to last five years from 1977, the intervention’s success saw its expansion to the rest of Finland and continuation until 2012. By 2012, North Karelia saw an 84% decrease in cardiovascular mortality and an 82% decrease across all of Finland. Like the Stanford Three Community Study, the North Karelia project demonstrated the importance of context-specific community participation in public health communication.

Community participation is critical to ensure that an intervention’s messages and materials resonate with potential audiences. There are many proven methods for designing and implementing communication interventions that seek to include community voices. For example, an intervention might seek to understand the needs of a given community through formative research and develop a health communication intervention based on those needs. Another feasible option is to build an intervention based on the inherent strengths and resources available in a community. Similarly, programs can be based on an ideology of building consensus among community members or can aim to question and restructure existing systems and practices. Accordingly, different approaches to including community voices can be based on a community’s needs and/or their existing strengths and can use consensus strategies that are based in collaboration and/or conflict strategies that are based in advocacy (5).

### 1.3 Community engagement in health communication

Community-based health communication interventions can be visualized as existing on a continuum. This idea has grown from Arnstein’s (6) Ladder of Citizen Participation, being often referenced as a tool for examining how much community participation, empowerment, and control exists in a given intervention. Measures of community participation do not label one approach as better than another, but instead aim to identify the best approach for a given context (7, 8). On one end, outside researchers or practitioners enter a community to help with a specific health issue and the community’s participation is limited to receiving the intervention. On the other end of the continuum are efforts that originate by and for the community and in which the community is an active participant during the implementation and reception of the project.

In a recent report by the World Health Organization (WHO), WHO reviewed relevant literature on community participation to clearly define four levels of community engagement, including community-oriented, community-based, community-managed, and community-owned (9). Each level of community engagement reflects a different balance of stakeholder involvement, with oriented interventions involving the greatest amount of external support and owned interventions involving the least. In community-oriented interventions, stakeholders are at least informed and included to participate in an intervention that aims to affect immediate, short-term change. Increasing the involvement of stakeholders, community-based interventions actively consult and involve the community in the improvement of health outcomes. Community-managed interventions require the collaboration of leaders who enable the community to set their own priorities and make autonomous decisions about the development, implementation, or evaluation of an intervention. At the highest end of stakeholder involvement and lowest level of external support, community-owned interventions ensure that a community has full ownership of the intervention at all stages (9). This review seeks to use the WHO definition of levels of community engagement as a standard continuum of evaluation.

There is much literature on the community engagement processes and health outcomes of interventions for specific issues such as maternal and child health (10, 11) or in specific contexts, such as rural health (12). These and other recent reviews examined the processes and outcomes of interventions that sought to support community participation, however they have shown mixed results (13–15). It has been suggested that these mixed results are in part due including community participation as an aspect of an intervention, instead of a process for long-term change. Because community participation is highly context-specific, approaching it as a standardized, short-term solution for promoting health instead of a tailored, long-term process for enacting social change can fail to create meaningful, long-lasting effects (13–15). In addition to being highly complex in an increasingly globalized society, social and political contexts are also dynamic. Considering this, addressing contextual differences in power and control has been highlighted as a necessary part of community participation (16).

This review seeks to better understand how “global” health communication programs tailor community engagement approaches and strategies to these unique contexts. “Global” health seeks to address transnational health issues and achieve health equity (17), so interventions that are situated in a “global” context must address similar issues (e.g., maternal health, noncommunicable disease, etc.) while being tailored to diverse local contexts. Therefore, a focus on “global” health communication interventions can illuminate how these interventions consider the intersecting identities, needs, and strengths of the communities they seek to engage with.

### 1.4 Community engagement strategies and approaches

Wallerstein et al. (5) reviewed several different types of community intervention models to create a typology of community interventions based on a combination of approaches and strategies, with the purpose of placing community in the center of public health practice. This model incorporates both needs-based and strengths-based approaches and describes strategies that are designed to build consensus or to use conflict for creating social action. Community organization and community building positions social and behavior change interventions as falling into four quadrants characterized by two broad approaches (strengths and needs) and encompassing two broad strategies (consensus and conflict). The purpose of this framework is to inform public health efforts that seek to engage communities, highlighting the importance of creating supporting environments that empower community participation and social action. Considering this, Wallterstein’s framework translates well to global health communication because of a need to both meet community needs and tailor interventions to unique, divergent social contexts that contribute to health inequities. By categorizing global health communication interventions into these quadrants, this paper attempts to shed light on the approaches and strategies used to foster different levels of community engagement, i.e., the level to which beneficiaries are engaged in the health communication interventions that are designed to promote their health and well-being.

Strengths-based programs can be explained by Wallerstein et al.’s (5) framework, which builds upon the communities existing strengths through community capacity, empowerment, critical consciousness, participation, and relevance, and/or health equity. Needs-based interventions on the other hand rely on the community’s needs and/or if an intervention fulfills a desirable need. When reviewing an intervention to see if it met the strengths-based and/or the needs-based criteria, it is also important to measure if it is utilizing a consensus approach, which is primarily using collaboration strategies or a conflict approach, where they position themselves as questioning the status quo. Programs that use conflict approaches primarily focus on advocacy strategies and efforts (5). Interventions are classified as using a consensus approach when the objectives and outcomes of the program rely on collaboration, cooperation, or participatory planning. Additionally, the consensus approach emphasizes building group identity and problem solving through community development, community building and capacity building (5). Interventions classified as using a conflict approach rely on advocacy as the driving force. Advocacy strategies are driven by empirical data and seek to bring about long lasting social change (5). Based on their goals and objectives, global health communication interventions can combine different strategies at different times of program development, implementation, and evaluation to achieve multiple outcomes.

### 1.5 Informing community engagement in global health communication

Because of the importance of context-specific community participation in global health communication, closely examining what community engagement strategies and approaches are used in different contexts can help inform the design and implementation of future interventions. Contextual factors like where the intervention is location (e.g., global north, global south), who the target audience is (e.g., adults, women, children), or the health issue of concern (e.g., maternal and child health, noncommunicable disease, etc.), to name a few, all affect which approaches and strategies are best suited for a given intervention. Considering the mixed results of community engagement in global health found by similar reviews (13–15), this review seeks to make a novel contribution by cross-analyzing community-based global health interventions based on the aforementioned factors and their community engagement strategies and approaches (5). To our knowledge, no such cross-analysis of community engagement strategies and approaches exists for health communication interventions in a global context. Through this approach, this review seeks to examine the current state of the field, outline lessons learned, and identify gaps in existing programming to help guide future interventions. Analyzing whether a health program builds upon audience strengths, needs, or both, and whether it attempts to work in consensus or conflict with existing power structures, can be used to understand and address discourses of health communication in different cultural settings and contexts and help tailor future policies and programs.

## 2 Methods

### 2.1 Search terms

Search terms were predefined before searching for literature on PubMed and Web of Science. Related and alternate spellings of search terms were joined by topic with an OR operation, including global health (global, international), health communication (health communication, health communications), programs (program, intervention, project, activity, strategy), community (community, stakeholder, stakeholders), and engagement (engagement, participation, collaboration, coalition, coalitions, partnership, partnerships, grassroots, involvement, empowerment, development, building, organizing, organising, organization, organisation, mobilizing, mobilising, mobilization, mobilisation, leadership, advocacy, social action, driven). Each group of search terms were then joined with an AND operation. Since this review seeks to include literature on “global” health interventions, including “global” and “international” as search terms purposely selects phrases that refer to global or international health (e.g., “these findings have implications for global health because…”) and does not necessarily limit the results to only interventions that span multiple countries. The same search terms were used for PubMed and Web of Science.

### 2.2 Eligibility criteria

Inclusion and exclusion criteria are outlined in Table 1. Studies were included if they described the implementation of a community-based health communication intervention. Interventions were deemed to be community-based if they leveraged some level of community engagement in their design, implementation, or evaluation. Studies were not included if they did not use any community engagement or if the intervention did not identify a specific community but focused instead on large unspecified audiences, for example mass media campaigns designed to influence the general public. Studies were only included if the article described an ongoing or completed implementation, whereas articles that only described program development or that conducted secondary analyses of interventions were discarded.

**Table 1:** Inclusion and exclusion criteria.

	Inclusion criteria	Exclusion criteria
Language	English	non-English
Publication date	2010 to 2023	Before 2010
Article type	Journal articles	Reviews Background articles Case studies Opinions, letters, editorials, etc. Conference proceedings Study protocols Expert panels
Intervention	Describes some form of a health communication intervention Describes an intervention that is community-based Describes the completed or ongoing implementation of an intervention	No intervention is described An intervention is described but is not community-based (e.g., mass media) An intervention is described but there is no completed or ongoing implementation

### 2.3 Literature search and sample selection

Two electronic databases (PubMed and Web of Science) were searched for relevant studies using the predefined set of search terms previously described. The search was conducted in March 2023. Because few search results were published before 2010, and in the interest of focusing on recent literature, manuscripts published before 2010 were excluded. Search results were downloaded and imported into Rayyan, an online review software (18). All duplicates were removed.

One coder (J.S.) reviewed all articles with titles starting with A – M and a second coder (L.A.) reviewed all articles with titles starting with G – Z. Overlapping articles between the two coders ensured interrater reliability. Each coder first reviewed each title to identify articles that could be easily excluded. The abstracts of each remaining article were reviewed for inclusion. Inter-rater reliability between the first two coders was quantified using Cohen’s kappa score; Cohen’s kappa scores above 0.80 demonstrate strong inter-rater reliability (19). A third coder (S.S.) reviewed all the shortlisted articles. Manuscripts were only included or excluded if at least two of the three coders made the same decision. Conflicting decisions were discussed and resolved by all three coders. Some additional articles were discussed and excluded from the analysis if they were deemed to not meet the eligibility criteria after the full-text review.

### 2.4 Coding

After all articles received at least two matching decisions among the three coders, a full-text review of included articles was conducted and input into an Excel matrix. Descriptive information about interventions was extracted when available, including the intervention’s name, region, years implemented, target audience, health issue, and conceptual framework. Regions were defined according to UNICEF’s regional classifications. Detailed data were also collected about each intervention’s communication channels, including modes and methods, and desired intermediate outcomes (e.g., knowledge, attitudes, beliefs, etc.), behavioral outcomes, and long-term outcomes (e.g., prevalence, structural changes, etc.). When a conceptual model, theory of change, or framework was reported in the design of an intervention, they were also categorized and recorded. If the article included an evaluation of the intervention, the evaluation’s methods and purpose (e.g., effectiveness, process, etc.) were also coded. Interventions were categorized on four levels of community engagement as defined by WHO, including community-oriented, community-based, community-managed, and community-owned (9).

Interventions were determined to be strengths-based based on the framework described by (5), whereby an intervention can build upon a community’s strengths through community capacity, empowerment, critical consciousness, participation and relevance, or health equity. During the full-text review, developing personal skills, and creating supporting environments from the WHO (9) literature were added, resulting in seven strengths-based constructs. Any intervention could leverage one or more of these seven concepts. Two statements, one about the intervention and one about the community, were created for each concept (Table 2). If at least one statement was true for any intervention, then it was deemed that that it leveraged a strengths-based approach.

**Table 2:** Strengths-based concepts adapted from Wallerstein et al. (5) and WHO (9).

Concept	Statements
Community capacity	The intervention builds on community strengths by involving organizations and leaders to enhance connections and social networks. Community members are actively involved in identifying and solving their issues and preparing to address future problems.
Empowerment	The intervention promotes co-learning and emphasizes the exchange of skills, knowledge, and capacity. Community members are challenging power structures to affect desired outcomes.
Critical consciousness	The intervention involves interactive listening, dialog, and action based on reflection or mentorship Community members are engaged in listening and dialogs that link root causes to tangible actions.
Participation and relevance	The intervention involves the community in all stages of engagement. Community members create their own agenda based on their own needs, power, and resources.
Health equity	The intervention addresses inequitable conditions that create health disparities. Community members are allocated resources that challenge inequitable conditions.
Developing personal skills	The intervention develops personal and social skills through information and education. Community members are exercising more control over their own health and environments.
Creating supportive environments	The intervention aims to change the community’s life, work, and leisure habits to improve health. Community members take action to sustain the viability of their environment to improve health.

Two statements were designed for each concept, and if at least one was true for an intervention, then it was deemed that the intervention leveraged that concept.

An intervention was categorized as needs-based if the authors clearly demonstrated a need for the intervention and the intervention was designed with that need in mind. While all interventions necessarily address a gap, an intervention could be coded as not needs-based if the intervention’s design was not informed by needs that were identified by the community itself. In this case, a strengths-based intervention that was not needs-based would instead be informed by the resources and capabilities of community members. Interventions did not have to be either needs or strengths based, and an intervention could utilize both a needs-based and strength-based approach. Consensus and conflict approaches help describe an intervention’s primary strategy (5). Interventions that leverage collaboration as a primary strategy were categorized as consensus, whereas interventions that leveraged advocacy as their primary strategy were categorized as conflict. At the same time, an intervention could utilize both or neither approach.

Interventions were categorized as single, hybrid, or complex, depending upon their topology of approaches and strategies used. Single interventions utilized one approach and implemented one strategy (strengths or needs and consensus or conflict). Hybrid interventions utilized two approaches and implemented one strategy (strengths and needs and consensus or conflict), or utilized one approach and implemented two strategies (strengths or needs and consensus and conflict). Complex interventions utilized all four elements. All included records and coded variables were imported into STATA MP 17.0 for quantitative analysis.

Direct quotes that were descriptive of coded variables, including the level of community engagement, needs- or strengths- based approaches, and consensus or conflict strategies, were recorded. Quotes related to community engagement were extracted if they were representative of the levels of stakeholder engagement and/or amount of external support present. Quotes demonstrating a needs-based approach were extracted if they demonstrated how a need was identified by community members. In the case of a strengths-based approach, quotes were matched with one or more strengths-based concepts (as in Table 2) that the quote demonstrated. Similarly, quotes related to the consensus or conflict strategies were extracted if they were indicative of collaboration, consensus building, or advocacy activities that were a part of the intervention. Qualitative analysis was conducted using direct quotes to identify similar or unique themes among studies that used similar approaches.

## 3 Results

### 3.1 Literature search

**Figure 1:**
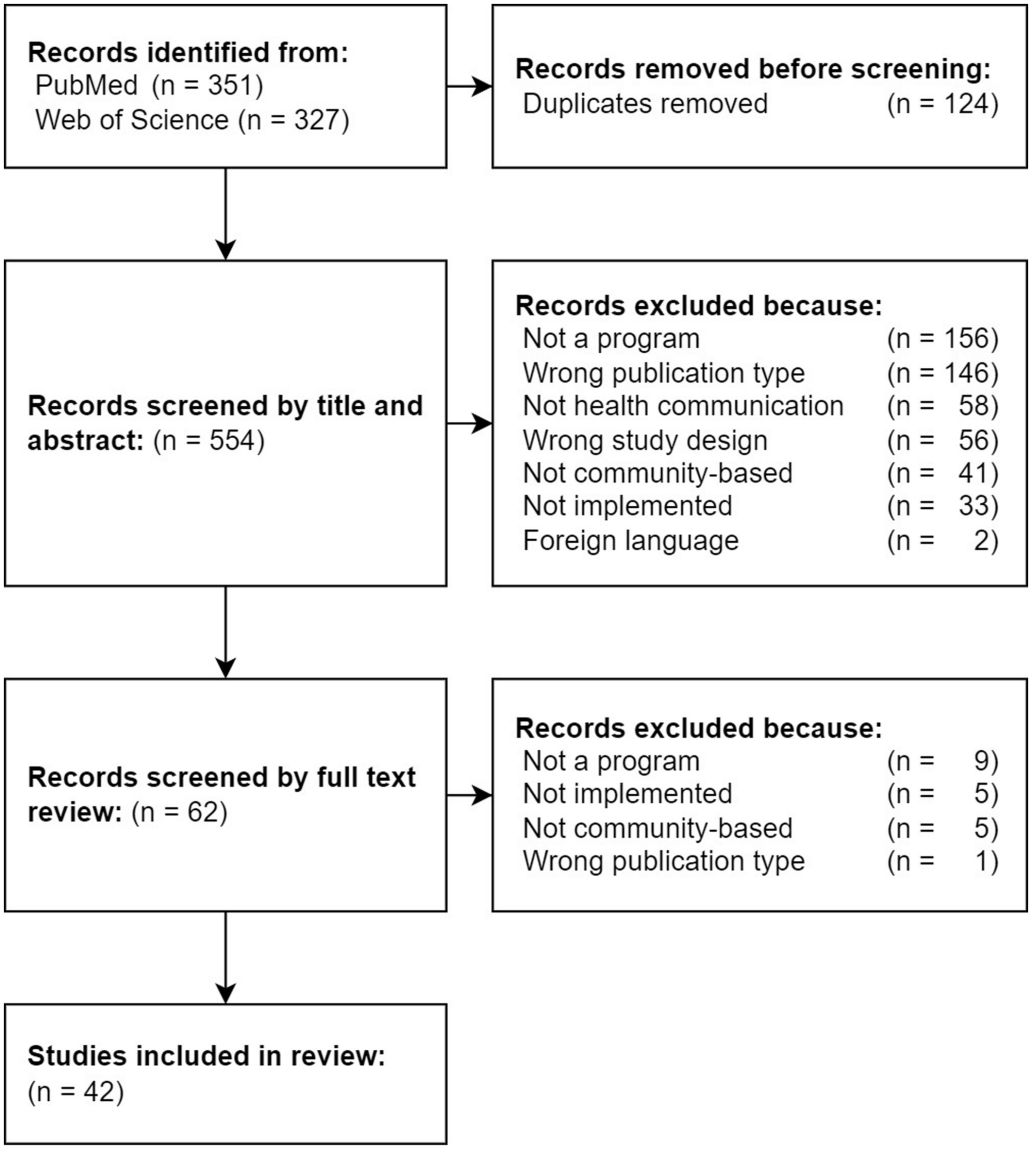
PRISMA flow diagram of all records returned from the literature search and reasons for exclusion during title, abstract, and full-text reviews.

The literature search yielded a total of 678 results, 351 from PubMed and 327 from Web of Science (Figure 1). Searching for duplicates excluded 124 records, and 554 were reviewed by their titles and abstracts. The full-text review included 62 records, from which 20 were excluded from this review. The final review and analysis included 42 studies. The Cohen’s kappa score between the first two coders (J.S. and L.A.) demonstrated strong inter-rater reliability (κ = 0.83). A complete summary of the key characteristics of every included study can be found in Supplementary Table S1.

### 3.2 Included studies

**Figure 2:**
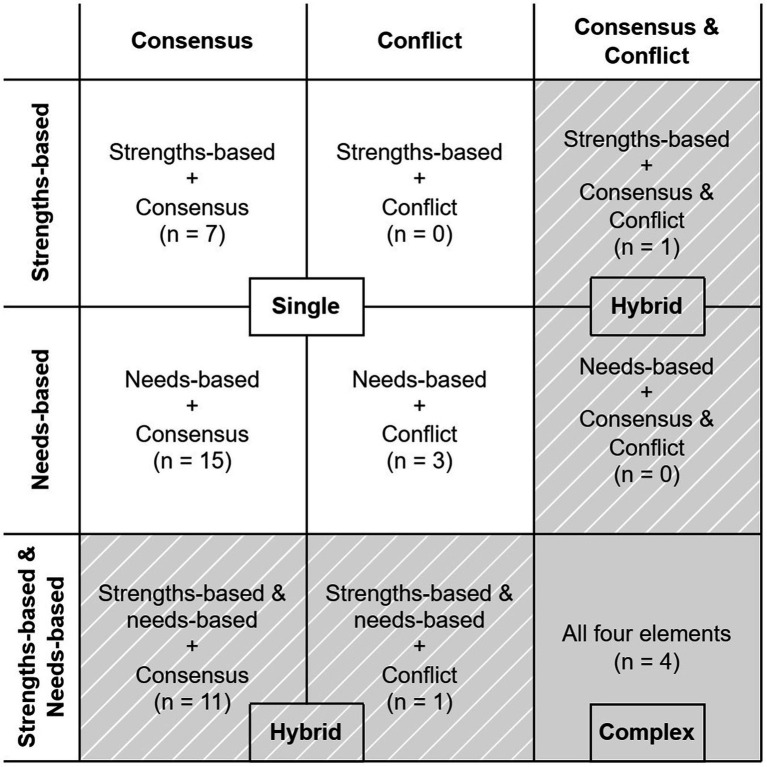
9 × 9 categorization of all interventions included in this study (*n* = 42) according to their approach (strengths, needs, or both) and strategy (consensus, conflict, or both). Single interventions include one approach and one strategy, Hybrid interventions include both approaches and one strategy or one approach and both strategies, and complex interventions include all four elements. The inclusion of hybrid and complex interventions extends the framework proposed by Wallerstein et al. (5).

The following sections will first report the descriptive results of the studies included in this review and of the approaches and strategies implemented in interventions. Interventions will also be reported according to the single, hybrid, and complex types (Figure 2). Examples of each level of community engagement will be provided for each type of intervention.

Table 3 summarizes the information pertaining to the descriptive elements of interventions that were gleaned from each included study. More than half of the studies reviewed described interventions that were implemented in North America or Eastern and Southern Africa, 12 (27.3%) articles in each region (Table 3). Among other regions, 6 (13.6%) interventions took place in West and Central Africa, 4 (9.1%) in South Asia, 3 (6.8%) in Western Europe, 2 (4.6%) in East Asia and Pacific, Latin America and Caribbean, and Middle East and North Africa each, and 1 (2.3%) in Australia/Oceania.

**Table 3:** Descriptive statistics of included studies.

	*N*%
Region	
East Asia and Pacific	1 (2.4)
Western Europe	3 (7.1)
Latin America and Caribbean	2 (4.8)
Middle East and North Africa	2 (4.8)
North America	11 (26.2)
South Asia	4 (9.5)
Eastern and Southern Africa	12 (28.6)
West and Central Africa	6 (14.3)
Australia/Oceania	1 (2.4)
Audience	
Adults	23 (54.8)
Children/youth	5 (11.9)
Healthcare workers	2 (4.8)
Racial/ethnic minorities	8 (19.1)
Other	4 (9.5)
Genders	
Men and women	27 (64.3)
Women only	9 (21.4)
Men only	1 (2.4)
Adolescents or children	5 (11.9)
Other (non-binary, transgender, etc.)	1 (2.4)
Health issue	
Sexual and reproductive health	9 (21.4)
Maternal and child health	9 (21.4)
Zoonotic disease	9 (21.4)
Noncommunicable disease	5 (11.9)
WASH	2 (4.8)
Health systems	4 (9.5)
Addiction	2 (4.8)
Other	2 (4.8)
Frameworks	
Participatory	5 (11.9)
Behavior change	7 (16.7)
Network/support	3 (7.1)
Cross-cutting	1 (2.4)
None	27 (64.3)
Communication channels	
Interpersonal communication	19 (45.2)
Community media/information sessions	18 (42.9)
Print media (posters, flyers, books, etc.)	14 (33.3)
Broadcast media (radio, television, etc.)	10 (23.8)
Interactive communication technologies	10 (23.8)
Level of community engagement	
Community-oriented	19 (45.2)
Community-based	12 (28.6)
Community-managed	8 (19.1)
Community-owned	3 (7.1)
Evaluation methods	
Quantitative (surveys, questionnaires, etc.)	25 (59.5)
Qualitative (interviews, focus groups, etc.)	24 (57.1)
Observation/physical measurement	12 (28.6)
Purpose of evaluation	
Impact/effectiveness	27 (64.3)
Formative	9 (21.4)
Process	18 (42.9)
Total	42 (100.0)

More than one gender, framework, communication channel, or evaluation method could be used in each intervention, so the rows in these categories are not mutually exclusive.

The primary audience of 23 (52.3%) interventions were adults, of 8 (18.2%) were racial/ethnic minorities, of 5 (11.4%) were children/youth, of 3 (6.8%) were healthcare workers, and of 1 (2.27%) were sexual/gender minorities. The primary audiences of 4 (9.1%) interventions were categorized as other vulnerable/at-risk populations, including people with Sexually Transmitted Infections, opioid use disorder, or cancer, or unhoused populations.

Close to two-thirds of the manuscripts 27 (64.3%) were aimed at both men and women and slightly less than a third of the manuscripts 15 (34.1%) described interventions aimed at one gender, only two manuscripts (designed specifically for sexual and reproductive health included three or more genders) (non-binary, transgender, etc.). While women were the audience for 9 (20.5%) interventions only one intervention was designed exclusively for men.

Among health issues, sexual and reproductive health, maternal and child health, and zoonotic diseases were each targeted by 9 (20.5%) interventions (Table 3). Noncommunicable diseases were targeted by 6 (13.6%) interventions. Health systems, like clinical trial participation (20) and healthcare accessibility (21), were targeted by 4 (9.1%) interventions. Addiction was targeted by 3 (6.8%) interventions and water, sanitation, and hygiene (WASH) by 2 (4.6%) interventions. Other health issues, including antimicrobial resistance (22) and household air pollution (23), were targeted by 2 (4.6%) interventions.

Only 15 (34.1%) interventions reported one or more conceptual models, theories of change, or frameworks that were used in their design (Table 3). Among 14 (%) studies that used frameworks the focus was on one specific level of the social ecological model: 6 (42.9%) used behavior change theories, 5 (35.7%) used participatory frameworks, 2 (14.3%) used network/support frameworks, and 1 (7.1%) used cross-cutting frameworks. Only one study in the sample reported using two categories of frameworks including both behavior change and a network/support framework.

One communication channel was used in 21 (47.7%) of interventions, two were used in 16 (36.4%) interventions, and three were used in 7 (15.9%) interventions. Interpersonal communication and community media/information sessions were the most used communication channels, each being used in 19 (43.2%) interventions. Print media was used in 15 (34.1%) interventions, broadcast media in 11 (25.0%), and interactive communication technologies in 10 (22.7%).

Based on the WHO (9) categorization, close to half the interventions were community-oriented, making up 21 (47.7%) of those included in this review (Table 3). Among other levels of community involvement, 11 (25.0%) interventions were community-based, 8 (18.2%) were community-managed, and 4 (9.1%) were community-owned.

Considering evaluation methods, 25 (56.8%) studies used one quantitative, qualitative, or observation/physical measurement evaluation method. Two methods were utilized in 18 (40.9%) studies and only one study used all three types of evaluation methods. A total of 27 (61.4%) studies used quantitative methods, 25 (56.8%) used qualitative methods, and 12 (27.3%) used some form of observation or physical measurement. Close to three-fourths of the studies (*n* = 32; 72.7%) had one purpose for their evaluation (impact/effectiveness, formative, or process) and 12 (27.3%) had two (Table 3). The purpose of 28 (63.6%) studies’ evaluations included impact/effectiveness, 18 (40.9%) included process, and 10 (22.7%) included formative.

### 3.3 Approaches and strategies

The frequencies of each approach and strategy, aggregated by the most common health issues and each level of engagement, are shown in Tables 4, 5. Of the strengths-based only interventions (*n* = 8), 2 (25.0%) interventions included maternal and child health issues, 3 (37.5%) included zoonotic diseases, and 2 (25.0%) included noncommunicable diseases. WASH, sexual and reproductive health, health systems, addiction and other health issues were included once or never among all strengths-based only interventions (Table 4). Of the needs-based only approaches (*n* = 18), 6 (33.3%) interventions included zoonotic disease, 5 (27.8%) interventions included sexual and reproductive health issues, and 4 (22.2%) interventions included maternal and child health issues. Noncommunicable diseases, WASH, health systems, addiction and other health issues were included once or never among all needs-based only interventions. Of strength and needs-based interventions (*n* = 16), 4 (25.0%) included sexual and reproductive health issues, 3 (18.8%) included maternal and child health and noncommunicable diseases, 3 (18.8%) included health systems and other health issues, with all other being included only once or never.

**Table 4:** Health issue and level of community engagement of interventions that used each approach.

	Strengths-based only	Needs-based only	Strength & needs-based
Health issue			
Sexual and reproductive health	0 (0.0)	5 (27.8)	4 (25.0)
Maternal and child health	2 (25.0)	4 (22.2)	3 (18.8)
Zoonotic disease	3 (37.5)	6 (33.3)	0 (0.0)
Noncommunicable disease	2 (25.0)	0 (0.0)	3 (18.8)
Level of community engagement			
Community-oriented	4 (50.0)	12 (66.7)	3 (18.8)
Community-based	3 (37.5)	4 (22.2)	5 (31.3)
Community-managed	0 (0.0)	1 (5.6)	7 (43.8)
Community-owned	1 (12.5)	1 (5.6)	1 (6.3)
Total	8 (100.0)	18 (100.0)	16 (100.0)

WASH, health systems, addiction, and other are excluded from this table because each have only a small sample size (*n* = 4 or less).

**Table 5:** Health issue and level of community engagement of interventions that used each strategy.

	Consensus only	Conflict only	Consensus & conflict
Health issue			
Sexual and reproductive health	6 (18.2)	2 (50.0)	1 (20.0)
Maternal and child health	4 (12.1)	2 (50.0)	3 (60.0)
Zoonotic disease	9 (27.3)	0 (0.0)	0 (0.0)
Noncommunicable disease	5 (15.2)	0 (0.0)	0 (0.0)
Level of community engagement			
Community-oriented	14 (42.4)	4 (100.0)	1 (20.0)
Community-based	10 (30.3)	0 (0.0)	2 (40.0)
Community-managed	7 (21.2)	0 (0.0)	1 (20.0)
Community-owned	2 (6.1)	0 (0.0)	1 (20.0)
Total	33 (100.0)	4 (100.0)	5 (100.0)

WASH, health systems, addiction, and other are excluded from this table because each have only a small sample size (*n* = 4 or less).

Community-oriented was the most frequently utilized level of community engagement (Table 4). A community-oriented level of engagement was implemented in 12 (66.7%) needs-based only interventions, 4 (50.0%) strengths-based only interventions, and 3 (18.8%) strengths- and needs-based interventions. A community-based level of engagement was used in 4 (22.2%) needs-based interventions, 3 (37.5%) strengths-based interventions, and was most commonly used in 5 (31.3%) needs- and strengths-based interventions. A community-managed level of engagement was implemented in 1 (5.6%) needs-based intervention and 7 (43.8%) strength and needs-based interventions. Community-owned was the least utilized among all levels, with only 1 intervention from each of all three approaches utilizing this level of community engagement.

Of the interventions that used consensus only strategies, 6 (18.2%) included sexual and reproductive health issues, 9 (27.3%) included zoonotic diseases, 5 (15.2%) included noncommunicable diseases, 4 (12.1%) included maternal and child health diseases, and all other health issues were studied only once or never (Table 5). Of the interventions that utilized conflict only strategies, only 2 (50.0%) included maternal and child health issues and 2 (50.0%) included sexual and reproductive health issues. Of the interventions that utilized consensus and conflict strategies, 3 (60.0%) included maternal and child health issues, and 1 (20.0%) included sexual and reproductive health and health systems issues.

Considering interventions that used a community-oriented level of engagement, the most used level, 14 (42.4%) consensus-only interventions were community-oriented, 4 (100.0%) conflict-only interventions were community-oriented, and 1 (20.0%) conflict and consensus intervention was community-oriented. Among consensus-only interventions, 10 (30.3%) included a community-based level of community engagement, 7 (21.2%) included community-managed, and 2 (6.1%) interventions included community-owned level of engagement. All conflict-only interventions were community-oriented. Among consensus and conflict interventions, community-oriented, managed, and owned levels were utilized by 1 (20.0%) intervention each, and 2 (40.0%) utilized a community-based level of engagement.

### 3.4 Types of interventions

Most interventions included in this review (34; 81.0%) utilized a needs-based approach and 24 (57.1%) utilized a strengths-based approach (Figure 2). Among single interventions (*n* = 25), 7 (28.0%) were strengths based and 18 (72.0%) were needs-based. Among hybrid interventions (*n* = 13), 1 (7.7%) was strengths-based only, while 12 (92.3%) were strengths- and needs-based.

Considering strategies, nearly all (38; 90.5%) interventions included in this review implemented a consensus strategy, compared to only 9 (21.4%) that implemented a conflict strategy. Among single interventions (*n* = 25), 22 (88.0%) implemented a consensus strategy only and 3 (12.0%) implemented a conflict strategy only. Among hybrid interventions (*n* = 13), 11 (84.6%) implemented a consensus strategy only, 1 (7.7%) implemented a conflict strategy only, and 1 (7.7%) implemented both consensus and conflict strategies.

Among all interventions included in this review, 4 (9.5%) were complex, being both strengths- and needs-based and implementing both strategies. No single interventions that implemented a conflict approach were strengths-based; all were needs-based. Conversely, no hybrid interventions that implemented a conflict strategy were needs-based only, but instead were strengths-based or strengths- and needs- based.

### 3.5 Single interventions

The following sections will describe studies that are representative of each intervention type. Within each section, examples of interventions are further sorted according to their level of community engagement (community-oriented, community-based, community-managed, and community-owned).

Among all interventions included in this review, 25 (59.5%) were single interventions, implementing one approach and one strategy. Of these 25 single interventions, 16 (64.0%) were community-oriented, 6 (24.0%) were community-based, 1 (4.0%) was community-managed, and 2 (8.0%) were community-owned.

#### 3.5.1 Community-oriented single interventions

Needs-based and consensus interventions sought to directly address community needs that were identified through previous or formative research. A consensus strategy implemented in these interventions emphasized collaboration or consensus-building through various methods. For example, an intervention described by Gamboa et al. (24) aimed to promote education and encourage preventative behaviors against arboviral diseases among the youth in the Dominican Republic. This intervention directly involved adults living in the areas where the intervention and evaluation took place. As a community-oriented intervention, community members were mobilized to address immediate and short-term needs related to prevention against arboviral diseases.

“Using Facebook as an education platform could impact knowledge and prevention behaviors related to arboviral diseases among youth living in communities at high risk for arboviruses within the Dominican Republic… The community leaders in this study consisted of adults living in the study areas who work in local public service organizations and had previously collaborated with the study team members.”

Conversely, needs-based and conflict approaches sought to meet an immediate need by challenging power structures or affecting change among those with authority over the target audience. For example, an intervention described by Dougherty et al. (25) aimed to shift social norms regarding maternal and child health in Ghana. Using a method of incentivization, this intervention involved all actors, including local leaders and authorities, to change traditional norms and structures. The community-oriented level of engagement leveraged by this intervention served to mobilize the community in addressing the community’s immediate needs.

“By incentivizing behavior change, [The Community Benefits Health program] aimed to encourage the entire community to support women in adopting improved maternal health and breastfeeding behaviors and swiftly change community-wide social norms by the end of the 2-year program.”

#### 3.5.2 Community-based single interventions

Another needs-based and consensus intervention described by Adam et al. (26) aimed to increase awareness and utilization of reproductive healthcare services among internally displaced women in Sudan. This intervention involved collaboration with community members who were familiar with the sociocultural context and directly addressed an immediate need for reproductive healthcare among displaced populations. This intervention was coded as community based because CHWs took part in disseminating information. On the ground, these CHWs could play a role in directing others to services in the community.

Compared to needs-based and consensus interventions, strengths-based and consensus interventions leveraged collaboration as a part of developing community strengths. For example, an intervention described by Tolentino et al. (27) aimed to increase awareness of COVID-19 prevention measures, like vaccines and mask wearing, awareness, and to combat misinformation and disinformation in Hawaii, USA. This intervention’s strengths-based approach empowered youth to challenge COVID-19 misinformation and disinformation by recognizing them as key leaders. Using a community-based level of engagement, this intervention consulted community organizations and involved youth to relay linguistically and culturally relevant information to diverse audiences.

“This strength-based approach recognized youth as important community leaders and ambassadors for change and empowered them to create content for dissemination on platforms with national and global reach… Next Gen Hawai‘i was founded to address these gaps from a strength-based approach, recognizing youth as important social media ambassadors and creative forces within their communities for public health and societal change.”

#### 3.5.3 Community-managed single interventions

Among single interventions, the only one that leveraged a community-managed level of engagement was a needs-based and consensus intervention. This intervention, described by Free et al. (28), aimed to improve individual behavior of high-risk sexual practices among people infected with chlamydia or gonorrhea. This intervention was coded as community-managed because its collaborative strategy involved community representatives who set priorities and made design decisions during the development, dissemination, and evaluation of the intervention.

#### 3.5.4 Community-owned single interventions

Another strengths-based and consensus intervention presented innovative strategies for including hard-to-reach or underrepresented populations. This intervention, described by Neugroschl et al. (29) aimed to increase health literacy regarding Alzheimer’s Disease among older adult Latinos in the US. As a strengths-based intervention, Neugroschl states that the process of giving community members a voice in the process of its development garnered buy-in from participants and ongoing collaboration with the study’s community advisory board. This intervention was coded as community-owned because this ownership ensured participation of community members in the development of a relevant intervention.

### 3.6 Hybrid interventions

Among all interventions included in this review, 13 (31.0%) were hybrid interventions, implementing two approaches and one strategy or one approach and two strategies. Of these 13 interventions, 2 (15.4%) were community-oriented, 5 (38.5%) were community-based, 6 (46.2%) were community-managed, and none were community-owned.

#### 3.6.1 Community-oriented hybrid interventions

Hybrid interventions that utilized strengths-based and needs-based approaches aimed to address community needs through strengths-based activities like building community capacity or critical consciousness. One that implemented a conflict strategy included advocacy efforts as a part of these activities. For example, an intervention described by Paek et al. (30) sought to improve family planning knowledge, attitudes, and behaviors in Ethiopia. This intervention addressed community needs related to maternal and child health through building on community strengths, developing community capacity and critical consciousness. The community-oriented level of engagement leveraged in this intervention involved collaboration with a nationwide organization that provided strong support to mobilize community members.

“[The Small, Happy, and Prosperous Family in Ethiopia (SHaPE)] employs integrated marketing communications and entertainment-education approaches with key messages that have been tailored for culturally specific audiences…Alongside its multi-media campaign, SHaPE includes two other components: (1) capacity building by training public health and media staffs and professionals; and (2) advocacy activities to build support that may affect policies.”

#### 3.6.2 Community-based hybrid interventions

Conversely, the hybrid intervention that implemented conflict and consensus strategies utilized an empowerment approach that promoted co-learning among those who took part. This intervention, described by Anderson et al. (31), used a public deliberation event to raise awareness of breastfeeding benefits and to garner support of breastfeeding in local businesses, encouraging participants to challenge existing power structures in the US. This article was coded as a community-based intervention as it ensured the community was consulted and directly involved to improve the awareness of breastfeeding and overall health programs with moderate external support.

“Grappling with the problem through public deliberation produced deep, personal involvement with the issue, which generates action. The conversations at the public deliberation event helped diminish the taboo of talking about breastfeeding, because they focused on clear, honest communication in a setting that fostered interpersonal relationships.”

Another community-based hybrid approach, described by Cueva et al. (32), aimed to improve cancer-related knowledge, attitudes, and beliefs among Indigenous populations in the US. This strengths-based and needs-based intervention implemented a consensus approach that used digital storytelling to create a culturally relevant and sustainable intervention. This filled a need for cancer-related education and supported consensus-building through digital storytelling with other community members. This intervention was coded as community-based because it directly consulted and involved community members in identifying and sharing stories.

#### 3.6.3 Community-managed hybrid interventions

Most of the hybrid interventions included in this review utilized strengths-based and needs-based approaches and implemented a consensus strategy. One intervention, described by Johnson et al. (33) built consensus through creating supportive environments to improve the community’s social or physical environment and improve health. This intervention aimed to reduce HIV risk factors like high-risk sexual behavior through extracurricular activities for primary school students in South Africa. After-school clubs discussed a television drama series. This approach served to transform students’ educational experiences by enabling them to take action in driving their own health behavior. Furthermore, this intervention addressed the community’s needs through a community-managed level. It collaborated with the schools and students through TV drama series and left the decision up to the people themselves for building positive health behavior.

“Children became [Soul Buddyz Clubs] members by engaging with special material and participating in activities, meetings and events which were run by trained Soul Buddyz facilitators. The facilitator provided support to the clubs but [Soul Buddyz Clubs] members drove the activities. The activities focused on building social support and community participation.”

Another community-managed intervention described by Figueroa et al. (34) sought to change sexual and gender norms related to HIV risk factors like high-risk sexual behavior among adults in Mozambique. Using a strengths-based and needs-based approach, the consensus strategy implemented by this intervention engaged participants in dialog with one another to promote collective education. For example, a radio broadcast included a segment allowing listeners to call in with concerns or questions. This form of two-way communication and other exchanges of ideas and peer mentorship empowered the community to set priorities and make decisions with only little external support.

### 3.7 Complex interventions

Only 4 (9.5%) of all interventions included in this review were complex, utilizing both strengths- and needs-based approaches while implementing both consensus and conflict strategies. Each complex intervention included in this review also utilized a unique level of community engagement, creating an equal spread of 1 (25.0%) complex intervention per level of community engagement.

#### 3.7.1 Community-oriented complex interventions

Complex interventions build upon community strengths to address immediate and long-term needs, while creating collaborative platforms that can aid health promotion and advocacy efforts. One complex intervention utilized a strengths-based and needs-based health equity approach to change inequitable conditions and address disparities in health. This intervention, described by Ndiaye et al. (35), allocated resources directly to affected communities to address health disparities. An Implementation Kit helped managers of other maternal and child health programs tailor their services to vulnerable clients. Using a community-oriented level of engagement, this intervention involved a high level of external support through financial support and technical assistance. This approach helped organize and mobilize communities to allocate resources and tailor interventions for their immediate needs.

#### 3.7.2 Community-based complex interventions

Another complex intervention utilized both strengths-based and needs-based approaches along with a conflict strategy to maximize the outcomes for the health issues. Adam et al. (36) aimed to influence a baby’s feeding patterns and change the method of delivery of health information from face-to-face to videos. It included both the need from the community and the gaps that existed as well as critical consciousness. Additionally, the intervention relied on a conflict strategy by advocacy and social change as its driving factor. This intervention was coded as community-based because it involved consultation with the community to address their needs. Community leaders also nominated peer mentors, which directly involved participants to improve access to health services by affecting behavior change related to maternal health.

#### 3.7.3 Community-managed complex interventions

A community-managed intervention described by Banerjee (37) detailed an online communication method that aimed to address vaccine hesitancy in rural India. Utilizing consensus and advocacy strategies, an online platform provided participants a space to collaborate and be involved in advocacy efforts. Furthermore, the level of community engagement leveraged by this intervention showcased the collaboration and prioritization of the decisions made by the participants to promote and address vaccine hesitancy. Faith leaders were involved throughout the duration of the intervention’s design and dissemination, enabling priority setting and long-term adoption by the community.

“A unique strength of this project was that it was designed to be implemented completely virtually as a result of the COVID-19 pandemic…This approach also allowed increased participation of community members and [community health workers] in the interventions, as they were able to join meetings from their homes without needing to travel long distances, limiting interference with their familial or professional responsibilities.”

#### 3.7.4 Community-owned complex interventions

Community-owned interventions empower the community to take part in the development and implementation of the intervention at all stages and strengthen networks through collaboration with external partners. A complex intervention described by Matsaganis et al. (38) aimed to improve reproductive healthcare utilization among African American women in the US. Beyond only priority-setting, this intervention also sought to build community capacity by strengthening existing networks and creating a sense of community ownership. Local community-based organizations were empowered to take a leading role in disseminating the intervention and promoting good health among community members.

## 4 Discussion

The purpose of this review was to explore current literature on global health communication programs to better understand how these programs tailored community engagement approaches and strategies to their unique contexts. A focus on “global” interventions examined those that were situated in contexts that required consideration of the intersecting identities, needs, and strengths of the communities they sought to engage. In an increasingly complex and interconnected world, community involvement is increasingly recognized as a process that is necessary to support long-term change (13–16). Therefore, to fulfill “global” health’s goal of addressing transnational health issues to achieve health equity (17), there is a need to recognize which community engagement strategies and processes are best suited for different contexts. The framework used in this review that was originally introduced by Wallerstein et al. (5) provided a method to closely examine how needs- or strengths-based approaches and consensus or conflict strategies were used for community engagement in different social and cultural contexts.

### 4.1 Geographical regions

Cross-analyzing the descriptive statistics reported in the results according to UNICEF’s regional classifications revealed differences between regions historically defined as the global north (North America, Western Europe, etc.) and global south (South Asia, West and Central Africa, etc.). Two thirds of the interventions were targeted at topics that required concerted community level action, for example zoonotic diseases or issues of maternal and child health. The interventions in regions historically classified as the global north were targeted toward race, ethnic, gender and sexual minorities. Out of 15 interventions from the global north, 12 dealt with minority populations, whereas 20 of the interventions from the global south were designed for a general health population. Several made mention of intersectionality, or a combination of social determinants of health that resulted in negative health outcomes.

Community interventions in regions historically classified as the global south tended to focus on the general population of adults. It is possible to hypothesize that these results display different connotations of the word community. In the global north, community refers to specific groups of individuals with a shared identity (American-Indians, homeless, cancer patients, health workers) whereas interventions from low- and middle-income countries define the general population as a community. Because this study sought to focus on “global” health communication, this difference may represent different applications of “global” health practice between geographical regions. In other words, when an intervention in the historical global north seeks to address transnational health issues and to achieve health equity (17), communities may be more narrowly defined according to shared identities, experiences, or actions (39). Conversely, communities may be more broadly defined when interventions in the historical global south seek to achieve the same goals. This difference could be in response to different cultural values and norms that constitute the meaning of community (40) or could indicate overgeneralizations of community membership that could marginalize those who are most vulnerable. However, stating this difference is due to either cultural differences or overgeneralizations of community membership would be an oversimplification of the issue. This geographical difference in the application of community in global health communication is an important question to consider for future research.

Different ways of describing communities based on geography likely have implications on the ways that health communication programs are conceptualized and implemented. Segmenting audiences into discrete groups allows for the generation of tailored messages with a focused cue to action. These types of interventions on the one hand are easier to design and implement in terms of human and financial resources. On the other hand, they may not resonate with core audiences who consider themselves to be outside of or on the periphery of the community being targeted. Additionally, when the level of community engagement in these interventions is community-oriented but largely led by experts who may not be a member of the targeted community, these interventions may lead to a lack of credibility and community ownership of the intervention.

### 4.2 Gender

The interventions also show a tendency to not disaggregate by gender, and when they do so, women tend to be the focus of these interventions. Not disaggregating by gender could have important implications. First, a lack of gender specificity in the interventions could fail to account for nuances in roles, responsibilities, and participation. In relatively traditional and collectivist communities where the power structures often disadvantage women and girls, female engagement could be limited by virtue of cultural and social norms. Second, on the other hand, when focused specifically on women, interventions fail to include men and boys, especially in reproductive, sexual, or maternal and child health (41), which are all areas where male engagement and involvement have shown to be successful strategies (42, 43). A tendency to not disaggregated by gender is likely a method employed by the included interventions for their specific contexts, and investigating the relationship between gender and an intervention’s approaches and strategies is an important area of future research.

### 4.3 Conceptual models

Only a third of the interventions reference a conceptual model or theory of change. The value of basing health communication interventions in theory is a universally accepted axiom. It is possible that the other interventions did not mention their use of theory in the actual manuscript being reviewed. Regardless, the lack of theoretical foundations in health communication interventions has been documented in other reviews from low- and middle-income countries (44). It is also notable that most of these interventions relied on individual level behavior change theories. While the need to keep individuals at the front and center of community efforts is often overlooked, this lack of cross-cutting theories or interpersonal or social theory constructs in intervention design and evaluation is noteworthy in its absence. Finally, given that community-based interventions often focus on face to face or virtual interaction with others, the fact that only two interventions relied on social support and social networks models is surprising. A limitation of this review is that the included studies may not report conceptual models or theories of change, even if they were used in the development of a given intervention. However, the reasons why group level theorizing is not being regularly reported as a basis of global health communication interventions that are grounded in communities is a question that remains to be answered.

### 4.4 Communication channels

The value of multi-channel and transmedia interventions to address public health issues is well documented in a wide range of interventions from addressing gender-based violence in Uganda to smoking cessation in the US and Canada (45). This review showed that close to half of the reviewed interventions used one communication channel. Given our focus on community interventions and the fact that traditionally communities have been described as location based, it is not surprising that the most used communication channels were interpersonal communication and community media/information sessions. On the other hand, mass media, including print and broadcast media, was also commonly used to disseminate information meant for communities. This is despite the fact that mass media interventions are generally considered to be most feasible for large populations. However, the fact that most interventions that employed mass media channels did so alongside other channels suggests that mass media can have an important role to play when employed in multi-channel interventions.

This review showed that 2 out of 10 reported interventions used some form of interactive communication technologies. Furthermore, the fact that reporting of interventions using these technologies is more common over time is proof of the growing importance of social media as a tool for community building. The different types of technologies used is in line with the current literature that recognizes that different forms of social media can serve both (top-down) institution centric and (bottom-up) sub-culture centric messaging (46).

### 4.5 Evaluation

In this review, more than half of the included studies used one evaluation method, the most common of which was quantitative measurement of effects. Surprisingly, relatively few studies described using evaluation as all stages of planning (formative), implementation (process) and evaluation (outcomes). Contrary to these findings, it would be expected that the formative research, monitoring, and evaluation of health communication interventions situated in communities would involve the collaboration of community members at all stages. For example, community based participatory research (CBPR) is a widely accepted principle for community collaboration (47). CBPR is a specific participatory research approach that empowers social change through cooperation, co-learning, and capacity building (48). These findings could reflect the reality that principles like CBPR are easier to talk about than it is to practice. There are several complex definitions as part of CBPR that require painstaking relationship building, collaboration, and learning across partnerships.

This need for comprehensive community involvement through principles like CBPR is reflective of similar recent reviews that highlight the importance of viewing community participation as a long-term process that is necessary for sustaining long-term change (10, 13–16). This review adds to the importance of this because of the divergent ways communities can be defined and the diverse social, cultural, and political contexts that are present in global settings. Considering CBPR, how a community defines itself vs. how others may define it, how participation itself is defined by different stakeholders in the research, and how research benefits and costs are perceived are just a few of the many complex considerations important to effective community engagement (49). Needs- and strengths-based approaches and consensus building or conflict strategies may aid the implementation of principles like CBPR in evaluation in these diverse contexts.

### 4.6 Strategies and approaches

The results from this review validated the Wallerstein et al. (5) framework that categorized interventions as using a strength or needs based approach and applied a conflict or consensus model to achieve their goals. However, this review found a fundamental weakness in simplifying intervention approaches and strategies into binary categories. Instead, we found health communication interventions fall under 9 different groups, often combining more than one approach or more than one model. These findings showcase the complex nature of health communication program planning and implementation, which require multiple implementation modalities. At the same time this review also highlighted homogeneity in health communication implementation, for example, needs and consensus were the most used strategies. The classification into nine categories with examples to illustrate these groupings indicates ways that health communication practitioners could broaden the scope of future interventions.

One of the most common topics in this review was zoonotic diseases, and therefore it is not surprising that needs as opposed to strengths was the most common approach used. Most zoonotic diseases require a rapid risk communication strategy and are concentrated on ensuring prevention or treatment and care for infected individuals. Comparing consensus and conflict strategies showed that 9 out of 10 interventions used consensus as a model. Overall, global health communication interventions based on community principles rely on identification of community needs and attempt to fulfill those needs through collaboration. Strengths based approaches when used are combined with consensus building with communities as well.

### 4.7 Hybrid interventions

As mentioned earlier, this review isolated several interventions which were hybrid in nature. These included implementing two approaches and one strategy or one approach and two strategies. There were only a handful of papers that combined both consensus and conflict with strengths or needs alone. Of the 13 interventions in this category, consensus building through community need identification while also capitalizing on their strengths is the most used approach. This group of hybrid interventions displays a positive trend. Global health communication has long been criticized for its pro-western bias. There are numerous examples of outside experts parachuting into countries with rigid ideas of what works and implementing them in local communities without regard for local context. Many health communication practitioners have moved away from a focus on external program design, implementation, and evaluation, to community-based approaches, where individuals with common interests coalesce around a specific health issue (5). This shift toward community-based approaches has happened based on consideration for unintended consequences of health communication campaigns that unintentionally modified the systems, values, and cultures of the society and its diverse subsectors (50). There is also the increased recognition of the role of culture which can be leveraged as a strength in health communication programs (51). Some scholars have described complex differences between individualistic cultures, that emphasize individual achievements and failures, and collectivist cultures, that emphasize community achievements and failures as western and eastern modes of communication (52).

According to Dutta et al. (53) a culture-centered approach to health communication addresses health disparities via participatory communication and authentic listening, especially to people who have been historically silenced and marginalized. By keeping both needs and strengths in focus, this culture centered approach focuses on the intersection of structure, culture, and agency (54).

### 4.8 Complex interventions

Another positive finding from this review is the four complex interventions that include all four elements. This could mean that the health communication literature is embracing the idea of complex public health interventions which are impacted by both upstream and downstream factors (55), An intervention can range in complexity based on several criteria – number of components, types of behaviors, the different social ecological levels that are addressed with varying expected outcomes and finally the extent to which interventions can be tailored (56). By adopting complexity science principles, global health communication interventions can be at the cutting edge of public health practice.

### 4.9 Levels of community engagement

Apart from categorizing global health communication interventions into nine typologies, this review also attempted to categorize each intervention by level of community participation. By further disaggregating the included interventions based on the WHO (9) model of levels of participation, this review also sought to explain if specific combinations of approaches and strategies were conducive to the level of community participation. The findings show interventions incorporating multiple approaches and strategies move from being community-oriented to community-owned.

Interestingly, needs-based interventions were more likely to be low on the participation spectrum and almost all were categorized as community-oriented. A needs-based approach does not automatically have to be ranked as community-oriented, health communication practitioners need to find innovative ways to craft interventions that are based on needs while being high on the community engagement spectrum. This is where principles like CBPR can play a vital role, involving communities in defining and ranking the problems that are central to them, allowing them to implement interventions in ways that are culturally contextualized and ascertaining their own metrics for success could be an important step in the right direction.

### 4.10 Conflict-based strategies

Planned health communication interventions tend to shy away from conflict-based strategies. One potential reason for the lack of interventions implementing conflict-based strategies could be the fact that much of the literature included in this review is grounded in the field of international development. The main focus of many of these interventions therefore is to improve the lives of individuals through collaboration and consensus. An emphasis on conflict as a strategy is more likely to be evident in examining social movements interested in correcting historical injustices, where marginalized communities are empowered to challenge power structures and achieve specific political and social goals (57). Since community participation is a long-term process for social change that can play an important role in addressing upstream factors, there is a need for empowering communities to challenge power structures (58).

A possible recommendation for interventions that seek to engage community members by implementing conflict-based strategies is social action theory (59). There is much to be learned from social movements that seek to enact change by empowering disadvantaged groups to have a voice, building support to address inequities and uphold the rights of individuals and communities, and/or mobilizing stakeholders toward a common cause. Scholars have described social movements as “communication movements” and highlighted the importance of addressing the communication components of social movements and the collective action that can result (60, 61). Insofar as self-efficacy and empowerment is the process to achieve health outcomes, this lack of using social action theory to highlight ways that communities could engage in simple doable social actions popularized by Saul Alinsky or exemplified in the Gandhian philosophy of non-cooperation as a health communication strategy requires closer attention (62, 63). There is a need to develop interventions that seek to empower communities to act on their own behalf, even if these actions are at odds with the existing power structures. Health communication implementers could focus on learnings of past social movements: demands for democracy in the Arab Spring, eco-feminism demonstrated by the Chipko (Hug the Trees) movement in India (64, 65). Many current social movements such as #METOO and #BLACKLIVESMATTER that demand sexual responsibility and bring to the public arena the relation between race and police brutality provide a real-time opportunity for health communication researchers interested in the social determinants of health to study conflict-based approaches to improved health and wellbeing.

The WHO categorization of community activities from being community-oriented to community-owned showed that the number of interventions using each level of engagement decreased as the level of engagement increased. Almost all of the interventions in this review are community-oriented, indicating that global health communication has a long way to go in its quest for being truly community-owned. Interventions in this review that employed hybrid or complex combinations of multiple strategies and approaches demonstrated a tendency toward higher levels of community engagement. Recognizing that public health interventions should be centered around their benefactors, closely examining which approaches and strategies should be used and when, can lead health communication toward community ownership. Through this, public health can play an important role in achieving the aforementioned goals of “global” health communication in an increasingly complex, interconnected world.

## Data availability statement

The original contributions presented in the study are included in the article/supplementary material, further inquiries can be directed to the corresponding author.

## Author contributions

JS, LA, and SS contributed to preparing the manuscript. JS and LA conducted the review and analysis with consultation with SS. All authors contributed to the article and approved the submitted version.
