# Supplementary material for: A review of strategies and levels of community engagement in strengths-based and needs-based health communication interventions
Source: Front Public Health. 2024 Apr 9;12:1231827. doi: 10.3389/fpubh.2024.1231827 (PMC11035763; doi:10.3389/fpubh.2024.1231827)
Supplement: Supplementary file 1 [file Table_1.docx]

Supplementary Table 1. Summary of articles included in this review.

|  | Name of intervention | Region | Audience | Health issue | Communication channels | Level of community engagement | Approaches and strategies |
| --- | --- | --- | --- | --- | --- | --- | --- |
| Adam et al. (1) |  | Eastern and Southern Africa | Racial/ethnic minorities  Women only | Sexual and reproductive health | Interpersonal communication  Community media/information sessions  Print media | Community-based | Needs-based  Consensus |
| Adam et al. (2) | Mentor-Mothers | Eastern and Southern Africa | Adults  Women only | Maternal and child health | Interpersonal communication | Community-based | Strengths-based  Needs-based  Consensus  Conflict |
| Anderson et al. (3) | Brookings Supports Breastfeeding | North America | Adults  Men and women | Maternal and child health | Community media/information sessions | Community-based | Strengths-based  Consensus  Conflict |
| Appiah et al. (4) |  | West and Central Africa | Adults  Men and women | Other | Community media/information sessions  Interpersonal communication | Community-managed | Strengths-based  Needs-based  Consensus |
| Banerjee et al. (5) | CIVIC Project | South Asia | Adults  Men and women | Systems | Broadcast media  Print media  Interactive communication technologies | Community-managed | Strengths-based  Needs-based  Consensus  Conflict |
| Brasington et al. (6) | SMART Intervention | Middle East and North Africa | Adults  Women only | Maternal and child health | Community media/information sessions  Print media | Community-oriented | Needs-based  Conflict |
| Cueva et al. (7) |  | North America | Racial/ethnic minorities  Men and women | Noncommunicable disease | Broadcast media  Print media | Community-oriented | Strengths-based  Consensus |
| Cueva et al. (8) |  | North America | Racial/ethnic minorities  Men and women | Noncommunicable disease | Interpersonal communication  Broadcast media  Interactive communication technologies | Community-based | Strengths-based  Needs-based  Consensus |
| Diez Canseco et al. (9) | The Multiplicadores Jóvenes (Young Multipliers) project | Latin America and Caribbean | Children/youth | Noncommunicable disease | Community media/information sessions | Community-based | Strengths-based  Needs-based  Consensus |
| Dougherty et al. (10) | The Community Benefits Health program | West and Central Africa | Adults  Women only | Maternal and child health | Community media/information sessions  Interpersonal communication  Broadcast media | Community-oriented | Needs-based  Conflict |
| Figueroa et al. (11) | Tchova Tchova Histórias de Vida: Diálogos Comunitários (Push Forward Life Stories: Community Dialogues) | Eastern and Southern Africa | Adults  Men and women | Sexual and reproductive health | Broadcast media  Community media/information sessions | Community-managed | Strengths-based  Needs-based  Consensus |
| Free et al. (12) | safetxt | Western Europe | Other vulnerable/at-risk populations (STIs)  Men and women | Sexual and reproductive health | Interactive communication technologies | Community-managed | Needs-based  Consensus |
| Gamboa et al. (13) |  | Latin America and Caribbean | Children/youth | Zoonotic disease | Interactive communication technologies | Community-oriented | Needs-based  Consensus |
| George et al. (14) |  | South Asia | Adults  Men and women | WASH | Interpersonal communication  Community media/information sessions  Broadcast media | Community-oriented | Needs-based  Consensus |
| Gutnik et al. (15) |  | Eastern and Southern Africa | Adults  Women only | Maternal and child health | Community media/information sessions | Community-oriented | Strengths-based  Consensus |
| Hildon et al. (16) | Tchova Tchova Stop Malaria | Eastern and Southern Africa | Adults  Men and women | Zoonotic disease | Interpersonal communication | Community-oriented | Needs-based  Consensus |
| Hove et al. (17) |  | Eastern and Southern Africa | Adults  Men only | Sexual and reproductive health | Interpersonal communication | Community-oriented | Needs-based  Conflict |
| Humeyestewa et al. (18) | Outreach, Health Education, Assessment for COVID-19,  Connection to Testing and Care | North America | Racial/ethnic minorities  Men and women | Zoonotic disease | Interpersonal communication  Print media  Broadcast media | Community-based | Needs-based  Consensus |
| Isac et al. (19) | Karnataka Health Promotion Trust | South Asia | Adults  Men and women  Other genders | Sexual and reproductive health | Interpersonal communication | Community-oriented | Needs-based  Consensus |
| Johnson et al. (20) | Soul Buddyz Clubs | Eastern and Southern Africa | Children/youth | Sexual and reproductive health | Community media/information sessions  Broadcast media | Community-managed | Strengths-based  Needs-based  Consensus |
| Kamhawi et al. (21) | Consult and Choose | Middle East and North Africa | Adults  Women only | Sexual and reproductive health | Print media  Interpersonal communication | Community-based | Needs-based  Consensus |
| Klouda et al. (22) | More Mobilizing Access to Maternal Health Services in Zambia | Eastern and Southern Africa | Healthcare workers  Men and women | Maternal and child health | Community media/information sessions | Community-oriented | Needs-based  Consensus |
| Lecerof et al. (23) | International Health Advisors | Western Europe | Racial/ethnic minorities  Men and women | Systems | Interpersonal communication | Community-owned | Needs-based  Consensus |
| Lefebvre et al. (24) | Communities That HEAL | North America | Other vulnerable/at-risk populations (opioid use)  Men and women | Addiction | Print media  Interactive communication technologies | Community-managed | Strengths-based  Needs-based  Consensus |
| Mamba et al. (25) | Community Driven Total FANC Attendance | Eastern and Southern Africa | Adults  Women only | Maternal and child health | Community media/information sessions | Community-oriented | Strengths-based  Needs-based  Consensus |
| Manana et al. (26) |  | Eastern and Southern Africa | Adults  Men and women | Zoonotic disease | Community media/information sessions | Community-oriented | Needs-based  Consensus |
| Matsaganis et al. (27) | Women's Health Project | North America | Racial/ethnic minorities  Women only | Sexual and reproductive health | Community media/information sessions  Interpersonal communication | Community-owned | Strengths-based  Needs-based  Consensus  Conflict |
| Meropol et al. (28) | Preparatory Education About Clinical Trials | North America | Other vulnerable/at-risk populations (cancer)  Men and women | Systems | Interactive communication technologies  Print media | Community-oriented | Needs-based  Consensus |
| Mitchell et al. (29) | Lurra RHD Project | Australia/Oceania | Children/youth | Noncommunicable disease | Print media  Interpersonal communication | Community-based | Strengths-based  Needs-based  Consensus |
| Namagembe et al. (30) |  | Eastern and Southern Africa | Adults  Men and women | Other | Interpersonal communication  Community media/information sessions  Print media | Community-managed | Strengths-based  Needs-based  Consensus |
| Ndiaye et al. (31) | Healthy Timing and Spacing of Pregnancies I-Kit | West and Central Africa | Adults  Men and women | Maternal and child health | Print media | Community-oriented | Strengths-based  Needs-based  Consensus  Conflict |
| Neugroschl et al. (32) | ¿Qué le  pasa a mi memoria? | North America | Adults  Men and women | Noncommunicable disease | Community media/information sessions | Community-owned | Strengths-based  Consensus |
| O'Keefe et al. (33) | Our Smallest Warriors, Our Strongest Medicine: Overcoming COVID-19 | North America | Racial/ethnic minorities  Men and women | Zoonotic disease | Print media | Community-oriented | Strengths-based  Consensus |
| Oku et al. (34) | ‘Communicatetovaccinate’ project | West and Central Africa | Healthcare workers  Men and women | Systems | Interpersonal communication  Broadcast media | Community-based | Strengths-based  Needs-based  Consensus |
| Paek et al. (35) | SHaPE | Eastern and Southern Africa | Adults  Men and women | Sexual and reproductive health | Broadcast media  Print media | Community-oriented | Strengths-based  Needs-based  Conflict |
| Parveen et al. (36) |  | South Asia | Adults  Men and women | Zoonotic disease | Community media/information sessions | Community-based | Needs-based  Consensus |
| Roess et al. (37) |  | West and Central Africa | Adults  Men and women | Zoonotic disease | Community media/information sessions  Interpersonal communication | Community-oriented | Strengths-based  Consensus |
| Scull et al. (38) | Media Detective Family Program | North America | Adults  Men and women | Addiction | Interpersonal communication | Community-based | Strengths-based  Consensus |
| Specht et al. (39) |  | Western Europe | Other vulnerable/at-risk populations (unhoused)  Men and women | Zoonotic disease | Interactive communication technologies  Print media | Community-oriented | Needs-based  Consensus |
| Swigart et al. (40) |  | West and Central Africa | Adults  Women only | Maternal and child health | Interactive communication technologies | Community-oriented | Needs-based  Consensus |
| Tolentino et al. (41) | Next Gen Hawaiʻi social media project | East Asia and Pacific | Children/youth | Zoonotic disease | Interactive communication technologies | Community-based | Strengths-based  Consensus |
| Zacher et al. (42) | Strong Heart Water Study | North America | Racial/ethnic minorities  Men and women | WASH | Interpersonal communication  Interactive communication technologies | Community-managed | Strengths-based  Needs-based  Consensus |

**References**

1. Adam, IF, Nakamura, K, Kizuki, M, Al Rifai, R, Vanching, U. Relationship between implementing interpersonal communication and mass education campaigns in emergency settings and use of reproductive healthcare services: evidence from Darfur, Sudan. *BMJ Open*. (2015) 5:e008285. doi: 10.1136/bmjopen-2015-008285

2. Adam, M, Job, N, Mabaso, B, Bärnighausen, T, Kuhnert, KL, Johnston, J, et al. “The Videos Gave Weight to Our Work”: Animated mHealth Videos and Tablet Technology Boost Community Health Workers’ Perceived Credibility in Khayelitsha, South Africa. *Qualitative Health Research*. (2022) 32:1273-1284. doi: 10.1177/10497323221091504

3. Anderson, J, Kuehl, RA, Mehltretter Drury, SA, Tschetter, L, Schwaegerl, M, Yoder, J, et al. Brookings supports breastfeeding: using public deliberation as a community-engaged approach to dissemination of research. *Translational Behavioral Medicine*. (2017) 7:783-792. doi: 10.1007/s13142-017-0480-6

4. Appiah, B, Asamoah-Akuoko, L, Samman, E, Koduah, A, Kretchy, IA, Ludu, JY, et al. The impact of antimicrobial resistance awareness interventions involving schoolchildren, development of an animation and parents engagements: a pilot study. *Antimicrobial Resistance & Infection Control*. (2022) 11:26. doi: 10.1186/s13756-022-01062-6

5. Banerjee P, Seth, R, Dhaliwal, BK, Sullivan, A, Qiayum, Y, Thankachen, B, et al. Vaccine acceptance in rural India: Engaging faith leaders as vaccine ambassadors. *Frontiers in Public Health*. (2022) 10. doi: 10.3389/fpubh.2022.979424

6. Brasington A, Abdelmegeid, A, Dwivedi, V, Kols, A, Kim, Y-M, Khadka, N, et al. Promoting Healthy Behaviors among Egyptian Mothers: A Quasi-Experimental Study of a Health Communication Package Delivered by Community Organizations. *PloS One*. (2016) 11. doi: 10.1371/journal.pone.0151783

7. Cueva M, Kuhnley, R, Slatton, J, Dignan, M, Underwood, E, Landis, K. Telenovela: an innovative colorectal cancer screening health messaging tool. *International Journal of Circumpolar Health*. (2013) 72. doi: 10.3402/ijch.v72i0.21301

8. Cueva M, Kuhnley, R, Lanier, A, Dignan, M, Revels, L, Schoenberg, NE, et al. Promoting Culturally Respectful Cancer Education Through Digital Storytelling. *International Journal of Indigenous Health*. (2016) 11. doi: 10.18357/ijih111201616013

9. Diez Canseco, F, Boeren, Y, Quispe, R, Chiang Prado, ML, Miranda, JJ. Engagement of adolescents in a health communications program to prevent noncommunicable diseases: Multiplicadores Jóvenes, Lima, Peru. 2011. *Preventing chronic disease*. (2015) 12. doi: 10.5888/pcd12.140416

10. Dougherty, L, Stammer, E, Derbile, E, Dery, M, Yahaya, W, Gle, DB, et al. A mixed-methods evaluation of a community-based behavior change program to improve maternal health outcomes in the upper west region of Ghana. *Journal of Health Communication*. (2018) 23:80-90. doi: 10.1080/10810730.2017.1414901

11. Figueroa, ME, Poppe, P, Carrasco, M, Pinho, MD, Massingue, F, Tanque, M, et al. Effectiveness of community dialogue in changing gender and sexual norms for HIV prevention: evaluation of the Tchova Tchova program in Mozambique. *Journal of health communication*. (2016) 21:554-563. doi: 10.1080/10810730.2015.1114050

12. Free, C, Palmer, MJ, McCarthy, OL, Jerome, L, Berendes, S, Knight, M, et al. Effectiveness of a behavioural intervention delivered by text messages (safetxt) on sexually transmitted reinfections in people aged 16-24 years: randomised controlled trial. *BMJ*. (2022) 378. doi: 10.1136/bmj-2022-070351

13. Gamboa J, Lamb, MM, de la Cruz, P, Bull, S, Olson, D. Using social media to increase preventative behaviors against arboviral diseases: a pilot study among teens in the Dominican Republic. *mHealth*. (2019) 5:30–30. doi: 10.21037/mhealth.2019.07.03

14. George, CM, Inauen, J, Perin, J, Tighe, J, Hasan, K, Zheng, Y. Behavioral determinants of switching to arsenic-safe water wells: an analysis of a randomized controlled trial of health education interventions coupled with water arsenic testing. *Health Education & Behavior*. (2017) 44:92-102. doi: 10.1177/1090198116637604

15. Gutnik, L, Moses, A, Stanley, C, Tembo, T, Lee, C, Gopal, S. From community laywomen to breast health workers: A pilot training model to implement clinical breast exam screening in Malawi. *PloS one*. (2016) 11:e0151389. doi: 10.1371/journal.pone.0151389

16. Hildon ZJL, Escorcio-Ymayo, M, Zulliger, R, Arias de Aramburú, R, Lewicky, N, Harig, H, et al. “We have this, with my husband, we live in harmony”: exploring the gendered decision-making matrix for malaria prevention and treatment in Nampula Province, Mozambique. *Malaria Journal*. (2020) 19:133–133. doi: 10.1186/s12936-020-03198-5

17. Hove J, Masimba, L, Murenje, V, Nyadundu, S, Musayerenge, B, Xaba, S, et al. Incorporating Voluntary Medical Male Circumcision Into Traditional Circumcision Contexts: Experiences of a Local Consortium in Zimbabwe Collaborating With an Ethnic Group. *Global Health Science and Practice*. (2019) 7. doi: 10.9745/GHSP-D-18-00352

18. Humeyestewa, D, Burke, RM, Kaur, H, Vicenti, D, Jenkins, R, Yatabe, G, et al. COVID-19 response by the Hopi Tribe: impact of systems improvement during the first wave on the second wave of the pandemic. *BMJ Global Health*. (2021) 6:e005150. doi: 10.1136/bmjgh-2021-005150

19. Isac, S, Ramesh, BM, Rajaram, S, Washington, R, Bradley, JE, Reza-Paul, S, et al. Changes in HIV and syphilis prevalence among female sex workers from three serial cross-sectional surveys in Karnataka state, South India. *BMJ open*. (2015) 5:e007106. doi: 10.1136/bmjopen-2014-007106

20. Johnson, S, Magni, S, Dube, Z, Goldstein, S. Extracurricular school-based social change communication program associated with reduced HIV infection among young women in South Africa. *Journal of Health Communication*. (2018) 23:1044-1050. doi: 10.1080/10810730.2018.1544675

21. Kamhawi, S, Underwood, C, Murad, H, Jabre, B. Client-centered counseling improves client satisfaction with family planning visits: evidence from Irbid, Jordan. *Global Health: Science and Practice*. (2013) 1:180-192. doi: 10.9745/GHSP-D-12-00051

22. Klouda, T, Green, C, Soyoola, M, Quigley, P, Kureya, T, Barber, C, et al. How a communication intervention in Zambia re-oriented health services to the needs of the least-supported. *Healthcare*. (2018) 6. doi: 10.3390/healthcare6030114

23. Lecerof, SS, Stafström, M, Emmelin, M, Westerling, R, Östergen, PO. Findings from a prospective cohort study evaluating the effects of International Health Advisors’ work on recently settled migrants’ health. *BMC Public Health*. (2017) 17:1-10. doi: 10.1186/s12889-017-4273-0

24. Lefebvre, RC, Chandler, RK, Helme, DW, Kerner, R, Mann, S, Stein, MD, et al. Health communication campaigns to drive demand for evidence-based practices and reduce stigma in the HEALing communities study. *Drug and alcohol dependence*. (2020) 217:108338. doi: 10.1016/j.drugalcdep.2020.108338

25. Mamba, KC, Muula, AS, Stones, W. Facility-imposed barriers to early utilization of focused antenatal care services in Mangochi District, Malawi–a mixed methods assessment. *BMC pregnancy and childbirth*. (2017) 17:1-8. doi: 10.1186/s12884-017-1631-y

26. Manana PN, Jewett, S, Zikhali, J, Dlamini, D, Mabaso, N, Mlambo, Z, et al. “Maskandi experience”: exploring the use of a cultural song for community engagement in preparation for a pilot Sterile Insect Technique release programme for malaria vector control in KwaZulu-Natal Province, South Africa 2019. *Malaria Journal*. (2021) 20. doi: 10.1186/s12936-021-03736-9

27. Matsaganis, MD, Golden, AG, Scott, ME. Communication infrastructure theory and reproductive health disparities: Enhancing storytelling network integration by developing interstitial actors. *International Journal of Communication*. (2014) 8:21.

28. Meropol, NJ, Wong, YN, Albrecht, T, Manne, S, Miller, SM, Flamm, AL, et al. Randomized Trial of a Web-Based Intervention to Address Barriers to Clinical Trials. *Journal of clinical oncology: official journal of the American Society of Clinical Oncology*. (2016) 34:469–478. doi: 10.1200/JCO.2015.63.2257

29. Mitchell, AG, Diddo, J, James, AD, Guraylayla, L, Jinmarabynana, C, Carter, A, et al. Using community‐led development to build health communication about rheumatic heart disease in Aboriginal children: a developmental evaluation. *Australian and New Zealand Journal of Public Health*. (2021) 45. doi: 10.1111/1753-6405.13100

30. Namagembe, A, Muller, N, Scott, LM, Zwisler, G, Johnson, M, Arney, J, et al. Factors influencing the acquisition and correct and consistent use of the top-lit updraft cookstove in Uganda. *Journal of health communication*. (2015) 20:sup1, 76-83. doi: 10.1080/10810730.2014.994245

31. Ndiaye, K, Portillo, E, Ouedraogo, D, Mobley, A, Babalola, S. High-risk advanced maternal age and high parity pregnancy: tackling a neglected need through formative research and action. *Global Health: Science and Practice*. (2018) 6:372-383. doi: 10.9745/GHSP-D-17-00417

32. Neugroschl, J, Sewell, MC, Umpierre, M, Rodriguez, R, Meyers, L, Kranes, S, et al. Elderly Latino community members make an educational video: an academic-community collaboration to promote memory evaluations. *International psychogeriatrics*. (2019) 31:989-995. doi: 10.1017/S1041610218001448

33. O'Keefe, VM, Maudrie, TL, Ingalls, A, Kee, C, Masten, KL, Barlow, A, et al. Development and dissemination of a strengths-based Indigenous children's storybook:“Our Smallest Warriors, Our Strongest Medicine: Overcoming COVID-19”. *Frontiers in Sociology*. (2021) 6, 611356. doi: 10.3389/fsoc.2021.611356

34. Oku, A, Oyo-Ita, A, Glenton, C, Fretheim, A, Ames, H, Muloliwa, A, et al. Perceptions and experiences of childhood vaccination communication strategies among caregivers and health workers in Nigeria: A qualitative study. *PloS One*. (2017) 12. doi: 10.1371/journal.pone.0186733

35. Paek, HJ, Kim, H, Cho, Y, Hong, W, Ko, W, Choi, H, Youn, Y, Choi, Y, Balew, G, Doh, Y. Rationale, design, and characteristics of the multimedia family planning campaign for a small, happy, and prosperous family in Ethiopia (SHaPE). *BMC Public Health*. (2018) 18. doi: 10.1186/s12889-018-5799-5

36. Parveen, S, Islam, MS, Begum, M, Alam, MU, Sazzad, H, Sultana, R, et al. It’s not only what you say, it’s also how you say it: communicating nipah virus prevention messages during an outbreak in Bangladesh. *BMC Public Health*. (2016) 16:1-11. doi: 10.1186/s12889-016-3416-z

37. Roess, AA, Di Peppi, R, Kinzoni, EA, Molouania, M, Kennedy, E, Ibata, SR, et al. Knowledge Gained and Retained from a Video-Centered, Community-Based Intervention for Ebola Prevention, Congo. *Journal of Health Communication*. (2017) 22. doi: 10.1080/10810730.2017.1377321

38. Scull, TM, Kupersmidt, JB, Weatherholt, TN. The effectiveness of online, family‐based media literacy education for substance abuse prevention in elementary school children: Study of the Media Detective Family Program. *Journal of Community Psychology*. (2017) 45. doi: 10.1002/jcop.21893

39. Specht, ASarma, N, Linzbach, T, Hellmund, T, Hörig, M, Wintel, M, et al. Participatory development and implementation of inclusive digital health communication on COVID-19 with homeless people. *Frontiers in Public Health*. (2022) 10. doi: 10.3389/fpubh.2022.1042677

40. Swigart, T, Hollowell, J, Remes, P, Lavoie, M, Murray, J, Belem, M, et al. Can health promotion videos ‘go viral’? A non-randomised, controlled, before-and-after pilot study to measure the spread and impact of local language mobile videos in Burkina Faso. *Global Health Action*. (2019) 12:1600858.

41. Tolentino, M, Millerd, S, Bali, NZ, Ranido, E, Takiguchi, J, Balaz, HJ, et al. Next Gen Hawai’i: Collaborative COVID-19 Social Media Initiative to Engage Native Hawaiian, Other Pacific Islander, and Filipino Youth. *Hawai’i Journal of Health & Social Welfare*. (2022) 81:201–208.

42. Zacher, T, Endres, K, Richards, F, Robe, LB, Powers, M, Yracheta, J, et al. Evaluation of a water arsenic filter in a participatory intervention to reduce arsenic exposure in American Indian communities: The Strong Heart Water Study. *Science of The Total Environment*. (2023) 862:160217. doi: 10.1016/j.scitotenv.2022.160217
